# An open-access dataset of naturalistic viewing using simultaneous EEG-fMRI

**DOI:** 10.1038/s41597-023-02458-8

**Published:** 2023-08-23

**Authors:** Qawi K. Telesford, Eduardo Gonzalez-Moreira, Ting Xu, Yiwen Tian, Stanley J. Colcombe, Jessica Cloud, Brian E. Russ, Arnaud Falchier, Maximilian Nentwich, Jens Madsen, Lucas C. Parra, Charles E. Schroeder, Michael P. Milham, Alexandre R. Franco

**Affiliations:** 1 Center for Brain Imaging and Neuromodulation, Nathan S. Kline Institute for Psychiatric Research, Orangeburg, NY USA; 2 Center for the Developing Brain, Child Mind Institute, New York, NY USA; 3 Department of Psychiatry, New York University Grossman School of Medicine, New York, NY USA; 4 Department of Biomedical Engineering, The City College of the City University of New York, New York, NY USA; 5 Departments of Psychiatry and Neurology, Columbia University College of Physicians and Surgeons, New York, NY USA

**Keywords:** Biomedical engineering, Neuroscience, Research data, Functional magnetic resonance imaging, Electroencephalography - EEG

## Abstract

In this work, we present a dataset that combines functional magnetic imaging (fMRI) and electroencephalography (EEG) to use as a resource for understanding human brain function in these two imaging modalities. The dataset can also be used for optimizing preprocessing methods for simultaneously collected imaging data. The dataset includes simultaneously collected recordings from 22 individuals (ages: 23–51) across various visual and naturalistic stimuli. In addition, physiological, eye tracking, electrocardiography, and cognitive and behavioral data were collected along with this neuroimaging data. Visual tasks include a flickering checkerboard collected outside and inside the MRI scanner (EEG-only) and simultaneous EEG-fMRI recordings. Simultaneous recordings include rest, the visual paradigm Inscapes, and several short video movies representing naturalistic stimuli. Raw and preprocessed data are openly available to download. We present this dataset as part of an effort to provide open-access data to increase the opportunity for discoveries and understanding of the human brain and evaluate the correlation between electrical brain activity and blood oxygen level-dependent (BOLD) signals.

## Background & Summary

Simultaneous collection of electroencephalography (EEG) and functional magnetic resonance imaging (fMRI) data is an attractive approach to imaging as it combines the high spatial resolution of fMRI with the high temporal resolution of EEG. Combining modalities allows researchers to integrate spatial and temporal information while overcoming the limitations of a single imaging modality^1,2^. Nevertheless, collecting multimodal data simultaneously requires specific expertise, and researchers must overcome various technical challenges to successfully collect data. Such challenges may limit its broader usage in the research community.

There are several technical challenges encountered when collecting imaging modalities simultaneously. With EEG, the main challenge is due to various sources of noise that impact the recorded signal. Gradient artifact is the most significant source of noise in simultaneous recordings, caused by the magnetic field gradients during fMRI acquisition, which induce current into EEG electrodes^3^. Another noise source is the ballistocardiogram (BCG) signal, which captures the ballistic forces of blood in the cardiac cycle^4,5^. The BCG artifact arises from the pulsation of arteries in the scalp that causes movement in EEG electrodes and generates voltage. The BCG artifact is more pronounced in a strong magnetic field and increases with field strength^6^. In addition to gradient and BCG artifacts, other noise sources include the MRI helium compressor^7^, eye blinks^8^, head movement, and respiratory artifacts^9^. Additionally, while collecting fMRI data, one of the main issues is patient discomfort while wearing the EEG cap in the scanner, which can cause increased head motion. Likewise, preparation time for collecting both datasets can also increase participant burden. Collecting simultaneous fMRI and EEG requires overcoming a variety of technical challenges but also needs advanced preprocessing techniques to overcome these unavoidable artifacts and produce a cleaner signal. In this paper, we detail how we addressed various technical challenges encountered when recording simultaneous EEG-fMRI including strategies to improve data quality.

For this dataset, most of the tasks performed by the participants are naturalistic viewing tasks. Naturalistic stimuli represent paradigms considered more complex and dynamic than task-based stimuli^10,11^. Naturalistic viewing provides more physiologically relevant conditions and produces closer to real-world brain responses^12–14^. Naturalistic stimuli also contain narrative structure and provide context that reflects real-life experiences^14,15^. Moreover, movies have been found to have high intersubject correlation and reliability^16,17^, hold subjects’ attention^18^, and improves compliance related to motion and wakefulness^19^. Naturalistic movies are also an ideal stimulus for multimodal data sets and may be useful in linking responses across levels^20,21^ and species^22^.

In this manuscript, we present a dataset collected at the Nathan S. Kline Institute for Psychiatric Research (NKI) in Orangeburg, NY, representing a study using simultaneously collected EEG and fMRI in healthy adults. The dataset contains multiple task conditions across two scans, including a visual task, resting state, and naturalistic stimuli. We also present quality control metrics for both modalities and describe preprocessing steps to clean up the EEG data. Lastly, we openly share these raw and processed data through the International Neuroimaging Data-Sharing Initiative (INDI) along with preprocessing code available on GitHub.

## Methods

### Participants and procedures

Simultaneous EEG-fMRI was collected in twenty-two adults (ages 23–51 years; mean age: 36.8; 50% male) recruited from the Rockland County, NY community. Participants enrolled in this study have no history of psychiatric or neurological illnesses. All imaging was collected using a 3 T Siemens TrioTim equipped with a 12-channel head coil. EEG data were collected using an MR-compatible system by Brain Products consisting of the BrainCap MR with 64 channels, two 32-channel BrainAmp MR amplifiers, and a PowerPack battery. Cortical electrodes were arranged according to the international 10–20 system. Inside the scanner, eye tracking was collected in the left eye using the EyeLink 1000 Plus.

Participants attended two sessions between 2 and 354 days between scans (time between scans, mean: 38.2 days; median: 11 days); see Table 1 for the breakdown of data acquired during sessions. The scanning protocol consisted of three recording settings. The “Outside” setting was an EEG recording collected outside the MRI scanner in a non-shielded room; the “Scanner OFF” setting consisted of EEG recordings collected inside the static field of the MRI scanner while the scanner was off; the “Scanner ON” setting consisted the simultaneous EEG and fMRI recordings. All research performed was approved by NKIs Institutional Review Board (IRB# 941632). Prior to the experiment, written informed consent was obtained from all participants. Participants also provided demographic information and behavioral data, including information on their last month of sleep (Pittsburgh Sleep Study)^23^, the amount of sleep they had the previous night, and their caffeine intake before the scan session.

**Table 1:** Simultaneous EEG-fMRI experimental design. EEG and fMRI were collected simultaneously across two scan sessions.

Session	Recording Location	Scan	Run Length (s)
Day 1	Outside	Checkerboard (12 Hz)	200
	Scanner OFF	Checkerboard (12 Hz)	200
	Scanner ON	Checkerboard (12 Hz)	200
		Rest	600
		Inscapes	600
		The Present [Run 1]	258
		PEER	90
		Monkey 1 [Run 1]	300
		Despicable Me (English) [Run 1]	600
		MPRAGE	600
		The Present [Run 2]	258
		Monkey 1 [Run 2]	300
		Despicable Me (English) [Run 2]	600
Day 2	Outside	Checkerboard (12 Hz)	200
	Scanner OFF	Checkerboard (12 Hz)	200
	Scanner ON	Checkerboard (12 Hz)	200
		Rest	600
		Inscapes	600
		Monkey 2 [Run 1]	300
		PEER	90
		Despicable Me (Hungarian) [Run 1]	600
		Monkey 5 [Run 1]	300
		MPRAGE	600
		Monkey 2 [Run 2]	300
		Despicable Me (Hungarian) [Run 2]	600
		Monkey 5 [Run 2]	300

### EEG acquisition

The entire procedure of data collection takes approximately three hours. About 45 minutes are spent preparing a participant for EEG. Preparation begins with measuring the participant’s head to determine cap size. An anterior to posterior measurement is also taken across the top of the head, from the nasion to the inion. A mark is made at the center of the forehead 10% of the measured nasion-inion distance. A proper sized cap is placed on the participant’s head with the Fpz electrode centered at the mark on the forehead. Once the subject is fitted with the EEG cap, electrodes are filled with electrolyte gel. In this study, EEG is collected using a customized cap to record 61 cortical channels, two electrooculogram (EOG) channels placed above (channel 64) and below the left eye (channel 63), and one electrocardiography (ECG) channel (channel 32) placed on the back. In addition, the cap also contains a reference and ground electrode. Electrodes were filled using V19 Abralyt HiCl electrode gel. Electrode impedance was recorded before every recorded run; to ensure good data quality, electrode impedance was kept below 20kOhm. EEG was recorded using BrainVision Recorder at a sampling rate of 5 kHz. After cap preparation, participants completed a single run of the flickering checkerboard experiment in the “Outside” scan condition viewing a 19-inch LCD monitor. After the Outside scan, a 3D scan of the participant’s head is collected to digitize the position of EEG electrodes. 3D scans were collected using a portable scanner, the Occipital Structure Sensor (Occipital Inc, Boulder CO), and iPad Mini 4 (Apple, Cupertino, CA). Due to protected health information (PHI) restrictions, 3D scans will not be available in the data release; however, location files containing the positions of the electrodes will be provided with this release.

### Simultaneous EEG-fMRI recording in the MRI scanner

After 3D digitization, participants enter the MRI scanner and are placed on the scanner bed in the supine position. Cushions are placed around the head to provide stabilization and minimize head motion during scans. At this stage, participants are provided with MR-safe goggles if they have any visual impairment that requires glasses. The screen is rear projected at the end of the MRI scanner at 1300 mm to the mirror mounted on the head coil. The videos are shown on a projector screen with a size of 440 × 330 mm with a 1024 × 768 resolution. This creates a horizontal and vertical viewing angle of 19.35° and 14.51° respectively, with a resolution of 0.0189 degrees/pixel for both directions. The light in the room was kept on during the imaging session. Participants were also fitted with a respiratory transducer belt to monitor breathing, which was recorded using BIOPAC MP150 (BIOPAC Systems, Inc., Goleta, CA).

**Fig. 1:**
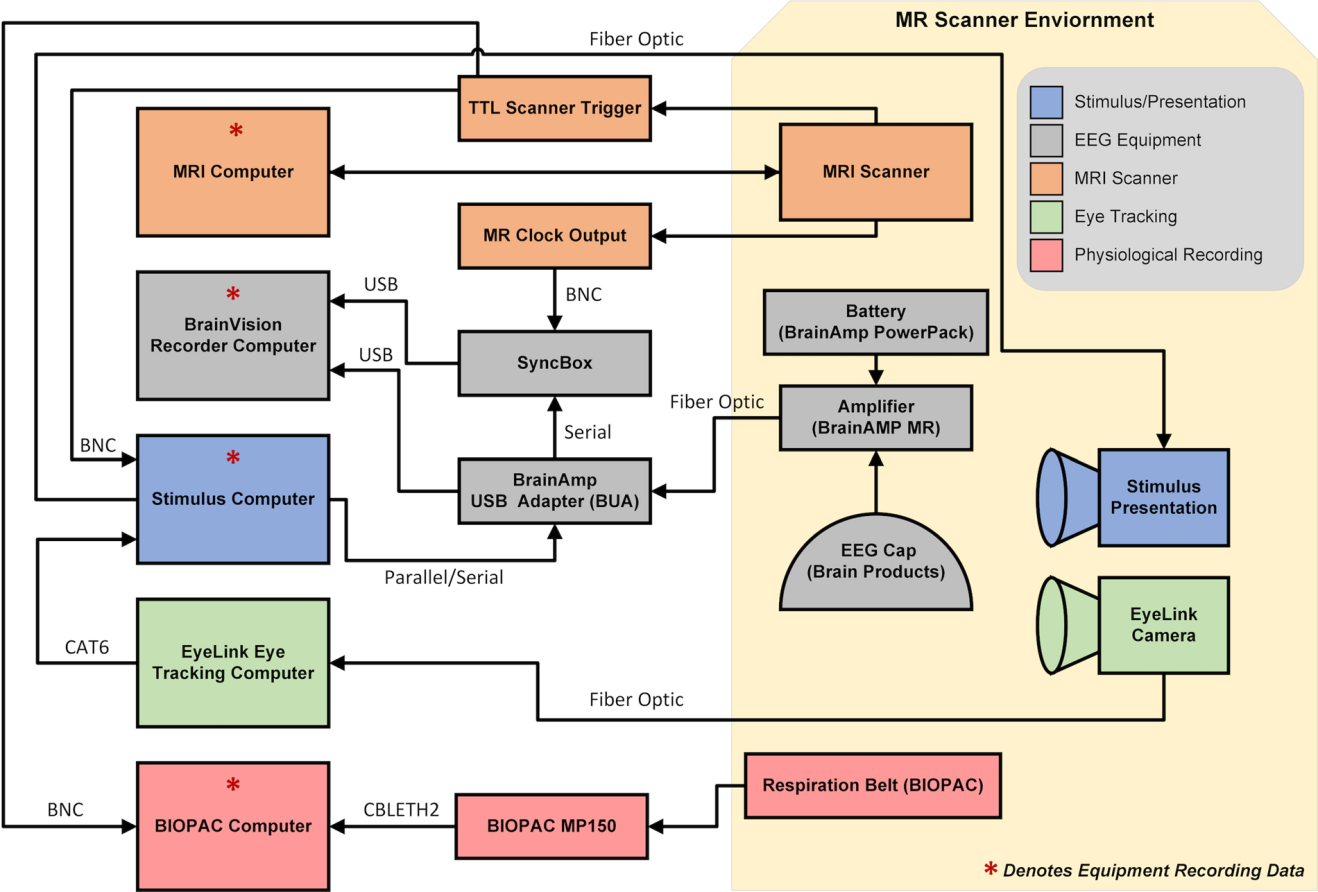
Schematic of EEG-fMRI setup.

Once positioned in the scanner, the participant’s head is enclosed in a 12-channel 3 T head matrix MR coil. The cable bundle (flat ribbon) from the EEG cap is routed through the front of the head coil and fixed at the top of the head coil using medical tape. The cable bundles are connected to the amplifiers and battery pack. During EEG data acquisition, the software is synchronized to the master clock of the MRI scanner. During recordings, EEG data was continuously collected along with task onset triggers, and volume triggers were recorded at the beginning of each TR. For specifics on the equipment and connections for simultaneous recordings, see Fig. 1 and Table 2.

**Table 2:** Equipment used for data collection and stimuli presentation in EEG-fMRI study.

Modality	Equipment	Sampling Rate	Additional Information
EEG/EOG/ECG	Brain Products BrainCap MR with Multitrodes	5000 Hz	64 channels • EEG: 61 cortical electrodes • EOG: 2 channels ○ Placed below and above left eye ○ EOGL (Lower: channel 63) ○ EOGU (Upper: channel 64) • ECG: 1 channel ○ placed on participant’s back below left shoulder blade (channel 32)
fMRI	Siemens 3.0 T TIM Trio	0.476 Hz (TR = 2100 ms)	12-channel head coil • TR = 2100 ms • TE = 24.6 ms • slices = 38 • matrix size = 64 × 64 • voxel size = 3.469 × 3.469 × 3.330 mm
Eye Tracking	EyeLink 1000 Eye Tracker	250 Hz or 1000 Hz	Left eye
Respiration	BIOPAC MP150	62.5 Hz	Placed around abdomen/chest
Stimulus Presentation	NEC LT220 DLP Projector	N/A	Video displayed at Display Resolution: 1024 × 768 Refresh Rate: 60 Hz
3D scans	Occipital Structure Sensor	N/A	3D scanner paired with Apple iPad Mini 4

### Eye tracking acquisition

For recordings collected inside the scanner, eye position and pupil dilation were recorded using an infrared-based eye tracker (EyeLink 1000 Plus, SR Research Ltd., Ontario, Canada; http://www.sr-research.com) at a sampling rate of 1000 Hz. Prior to release, data were down sampled to 250 Hz. The eye tracker was calibrated using a 9-point grid before recordings in the MRI scanner. Participants were asked to direct their gaze at dots presented on the grid. Calibration was followed by a validation step until the error between the two measurements was less than 1° ^24^.

### MRI data acquisition

MRI data were acquired using a 12-channel head coil on 3.0 T Siemens TIM Trio. MPRAGE structural T1w images were acquired with the following parameters: TR = 2500 ms; TI = 1200 ms; TE = 2.5 ms; slices = 192; matrix size = 256 × 256; voxel size = 1 mm^3^ isotropic; flip angle = 8°; partial Fourier off; pixel bandwidth = 190 Hz/Px. All BOLD fMRI sequences were acquired with these parameters: TR = 2100 ms; TE = 24.6 ms; Flip Angle = 60°; slices = 38; matrix size = 64 × 64; voxel size = 3.469 × 3.469 × 3.330 mm. The run length for each task is listed in Table 1.

### Task data/stimuli description

In this section, we describe the data generated for this study focused on collecting simultaneous EEG and fMRI. This dataset consists of a task, naturalistic stimuli, and resting state data. In addition to the simultaneously collected data, task data was collected outside the MRI scanner and inside the scanner environment with the scanner off. The collection of this data enables us to assess the impact of changes in the scanning environment on the EEG recordings. EEG-fMRI data were collected across two scan sessions; structural data was collected during the middle of the scan (see Table 1 for details). Code for presenting task stimuli and naturalistic stimuli, along with code to preprocess EEG and fMRI imaging data is available on GitHub (https://github.com/NathanKlineInstitute/NATVIEW_EEGFMRI).

#### Checkerboard stimulus

The use of flickering visual stimuli has been used to investigate the visual system in EEG^25,26^ and fMRI^27,28^. A high-contrast flickering checkerboard was used to stimulate the primary visual cortical regions. Participants were shown a flickering radial checkerboard at a frequency of 12 Hz in 20-second trials following a 20-second rest period across five repetitions. The checkerboard stimulus was presented outside the scanner after cap placement, inside the scanner with the scanner off, and inside the scanner during a simultaneous EEG and fMRI. These three recordings were collected to measure the impact of the MRI scanning sequence on the recorded EEG.

#### Rest

The participant is presented with a white fixation cross in the center of a black screen and instructed to rest with eyes open. Participants had one rest scan per session, and each scan had a duration of 10 minutes.

#### Inscapes

Inscapes is a computer-generated animation featuring abstract 3D shapes and moves in slow continuous transitions. The video was originally developed as a 7-minute video for children to watch during brain scans as a means to provide stimulation to keep them engaged while minimizing some cognitive processing that may be engaged^19^. Participants were presented with an extended version of Inscapes that was 10 minutes long. Similar to the rest scan, the Inscapes video was viewed once per scan session.

#### Predictive eye estimation regression (PEER) calibration

Predictive eye estimation regression (PEER) is an imaging-based calibration scan used to estimate the direction of eye gaze^29^ and here can be used as a complement to the optical eye tracking of the Eyelink 1000. Participants were told to direct their gaze at dots that appeared at predefined points on the screen. The PEER method estimates eye gaze from the collected scan using support vector regression (SVR). The algorithm estimates the direction of eye gaze during each repetition (TR) in the fMRI time series.

#### Naturalistic stimuli (Movies)

Participants viewed three movies twice in one scan session. Videos varied between 258 s and 600 s. Naturalistic stimuli included “The Present” [4m18s] (uploaded to YouTube 7 Feb 2016)^30^, two 10-minute clips from “Despicable Me” [clips taken from Russian Blu-Ray with exact times 1:02:09-1:12:09 (English) and 0:12:12-0:22:12 (Hungarian)]^31^, and three 5-minute monkey videos^32^. The monkey videos are part of a database with multiple videos^33^; for this study, Monkey 1, Monkey 2, and Monkey 5 represent the first, second, and fifth videos in the database, respectively. The videos used in this study are available to download in the GitHub repository (https://github.com/NathanKlineInstitute/NATVIEW_EEGFMRI/tree/main/stimulus).

### Limitations

The fMRI imaging in this dataset was collected using a 12-channel head coil on a 3 T scanner platform. While we possess the ability to scan at 3 T with a 32-channel head coil, the head coil design does not allow for the EEG cap cable bundle to be routed perpendicularly from the top of the head. Moreover, the design of the head coil does not permit alternate routing of the cable bundle because it will block the participant’s eyes or the cable length is too short to reach the amplifiers. In this study, the cable bundle is routed through the front and then taped to the top of the head coil before it is connected to the amplifier. Using a 12-channel head coil limited the sequences that could be used to collect fMRI, including the use of multiband sequences. Another limiting factor is that imaging sequences with faster TRs could not be used while collecting data simultaneously. The main issue is a safety concern regarding radiofrequency (RF) power deposition that causes heating in the EEG leads and electrodes during a sequence^34^. In this study, we used a TR of 2100 ms to ensure participants were not at risk of discomfort or burns due to the heating of electrodes.

We have not been able to calculate the delay between the stimulus computer and the projector, due to lack of equipment to conduct the appropriate measurement (photodiode)^35^. If in the future we do purchase this equipment and are able to perform this measurement, this information will be posted on the study website.

### EEG preprocessing

We developed an automated pipeline to preprocess all EEG data collected outside and inside the MRI scanner. The preprocessing methods used on the EEG data depended on where data were acquired. All EEG data were preprocessed using EEGLAB and associated plugins^36^. For data collected inside the scanner, data were preprocessed using the FMRIB plug-in for EEGLAB, provided by the University of Oxford Centre for Functional MRI of the Brain (FMRIB)^37,38^.

For data collected in the Outside setting, the following preprocessing steps were used: (i) bandpass filter using a Hamming windowed sinc FIR filter between 0.5 Hz and 70 Hz; (ii) reference electrodes using average reference, excluding the ECG channel, EOG channels, and electrodes excluded during the EEG quality control process.

For data collected in the Scanner OFF setting, the initial preprocessing steps used in the Outside setting were used. In addition, pulse artifact detection and removal were used due to the increased contamination of the signal caused by the participant’s heartbeat^3^: (i) QRS/heartbeat detection using the ECG channel; (ii) pulse artifact/BCG removal using template subtraction based on the median artifact; (iii) bandpass filter using a Hamming windowed sinc FIR filter between 0.5 Hz and 70 Hz; (iv) reference electrodes using average reference, excluding the ECG channel and EOG channels.

For data collected in the Scanner ON setting, the initial preprocessing steps used in the Scanner OFF setting were used. In addition, gradient artifact removal was used to remove the contamination of the EEG signal caused by the changing gradients from the fMRI pulse sequence^37^: (i) gradient artifact removal; (ii) QRS/heartbeat detection using the ECG channel; (iii) pulse artifact/BCG removal using template subtraction based on the median artifact; (iv) bandpass filter using a Hamming windowed sinc FIR filter between 0.5 Hz and 70 Hz; (v) reference electrodes using average reference, excluding the ECG channel and EOG channels.

#### Gradient artifact removal

The gradient artifact is the most significant noise source in simultaneous EEG-fMRI data, measuring more than 400 times larger than the lowest amplitude EEG events^3^. The FMRIB plugin uses the FASTR method to remove gradient artifacts^38^. The method requires recording the scanner trigger at the start of each TR. An average template is computed from the detected TRs and subtracted from the raw EEG data. Following this process, the data is corrected further using principal component analysis (PCA) to reduce residual artifacts. Residual artifacts are further reduced using adaptive noise cancellation^3^.

#### Pulse artifact/BCG removal

ECG data collected inside the MRI scanner has a pronounced T wave that increases as the field strength increases^39^. The FMRIB plugin identifies these QRS events using an algorithm that detects events, aligns them, and corrects for false positives and negatives^40,41^. A median signal is computed from the events to create an artifact template, which is subsequently subtracted from the data.

### MRI preprocessing

We used the Connectome Computation System (CCS) to preprocess the MRI/fMRI data^42^. For the anatomic data, we performed skull stripping using a combination of Brain Extraction Toolbox (BET) and Freesurfer. Data was then segmented (Freesurfer) and registered to a template space (MNI152 2006) using FLIRT and MCFLIRT^43,44^. The runs for the fMRI data were all preprocessed equally. Initially, the first five volumes are discarded, then the data is despiked and slice time and motion corrected. The functional data is skull striped with 3dAutomask, refined using the structural data, and registered to the anatomical images using boundary-based registration based on N4 using Freesurfer. Nuisance correction is done using the Friston 24 motion parameters, average CSF, and WM signals, with/without global signal regression (GSR). The data is also processed with/without temporal filtering (0.01–0.1 Hz) and with/without a 6 mm FWHM spatial filter. Time series were extracted from 400 ROIS (Schaefer 400 atlas) to be further processed^45^.

### Data quality control

#### EEG

To assess the quality of the EEG data in this study, we followed the quality control pipeline similar to that described in Delorme *et al*.^46^. This approach yields three metrics related to data quality: (i) percent of “good” channels; (ii) percent of “good” trials; and (iii) number of independent components (ICs) related to brain source activity. From this pipeline, “good” channels are defined as those that remain after completing the related preprocessing steps: removal of channels with more than five seconds of non-activity, with signal greater than four standard deviations due to high-frequency noise, or Pearson’s correlation coefficient less than 0.7 with nearby channels. Accordingly, “good” trials are related to the data periods that are not contaminated by artifacts such as body movement. In this study, we removed data segments with a variance higher than twenty times the variance of the calibration data. Finally, independent component analysis (ICA) was computed using the RunICA plugin for the EEGLAB toolbox to produce ICs^47^. Afterward, the ICLabel plugin was used to identify ICs belonging to brain source activity^48^. The resulting metric calculates the percentage of ICs associated with brain source activity divided by the total number of ICs found from ICA.

#### MRI

Temporal measures of fMRI data include median and median framewise displacement^44^, root mean square of the temporal change (DVARS), and temporal signal-to-noise ratio (tSNR).

### Statistical analysis

Permutation testing offers a robust framework for statistical significance assessment in EEG analysis. Multiple permutation testing was performed on the flickering checkerboard task to identify differences between the two task conditions: the rest and flickering checkerboard blocks. This analysis is used to find statistical relevance that identifies differences underlying the EEG data. One advantage of multiple permutation testing is that it does not require the same number of trials for each condition. In our permutation testing, trials were shuffled into two new groups, followed by the calculation of a paired t-test. Finally, pixel-based multiple comparisons correction was applied to reduce the familywise error rate^49^.

### Data privacy

All imaging data in this release has been de-identified, removing any personal identifying information (as defined by the Health Insurance Portability and Accountability) from data files, including facial features. Facial features from the T1w MRIs were removed using the “mri_deface” software package developed by Bischoff-Grethe *et al*.^50^. Data and code are shared under the CC BY 4.0 license.

## Data Records

### Data access

All data can be accessed through the 1,000 Functional Connectomes Project and its International Neuroimaging Data-sharing Initiative (FCP/INDI)^51^. This website^51^ provides directions for users to directly download all the imaging data from an Amazon Simple Storage Service (S3) bucket. Raw and preprocessed data is provided through the website.

### Data organization

All data have been organized following the Brain Imaging Data Structure (BIDS) format^52,53^, which is an increasingly popular approach to describing imaging data in a standard format. Within the study S3 bucket, there are two folders, one containing raw data and another containing preprocessed data.

Inside the raw data folder, the BIDS standard is followed, and the base folder contains five sets of files (JSON and TSV) with information about the study. This included a description of the dataset, a list of participants including demographic information, and questionnaires regarding sleep patterns, caffeine intake, and thoughts and feeling during the experiment^54^. Inside the subject and session folders, there are three folders: one containing the MRI anatomical images (anat), one containing the EEG and eye tracking data (eeg), and one file containing the fMRI and respiratory data (func). MRI data is stored in NIfTI format, EEG data is in EEGLAB data file format (.set) with header and marker files using Brainvision (.vhdr.vmrk) format, and eye tracking and respiratory data are stored in TSV’s. Sidecars (JSON files) with metadata are provided and contain information on acquisition parameters.

EEG and FMRI processed are stored in the preprocessed folder. Data is similarly organized as the raw data folder and file formats are the same. Completely preprocessed fMRI data are located in the “func_preproc” within each subject/session/func/task folder. Preprocessed EEG and eye tracking data are in the “eeg” folder within the subject/session subfolders.

## Technical Validation

### EEG data validation and quality

**Fig. 2:**
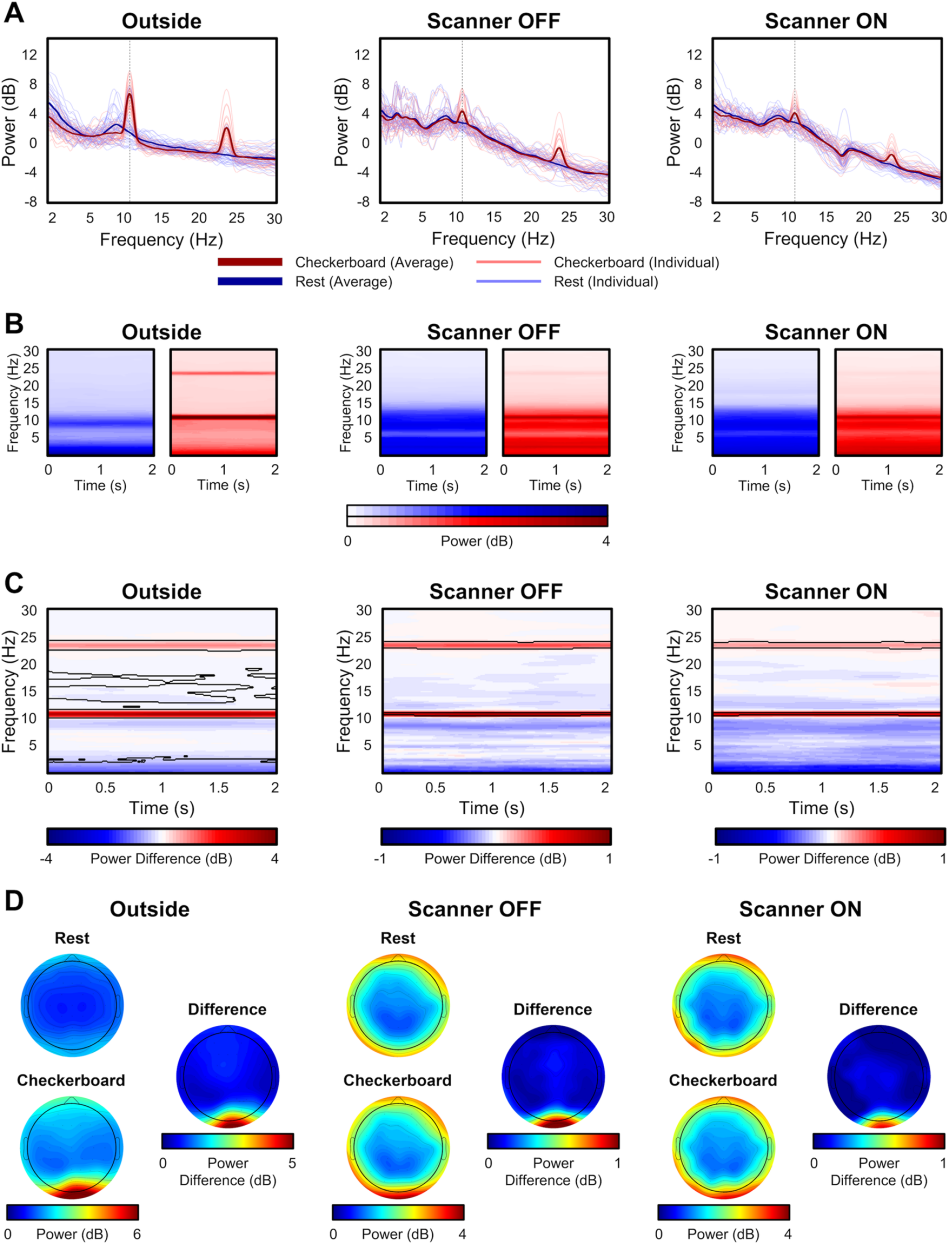
EEG data validation. (**A**) Comparison of the power spectrum for participants across scanning conditions: Outside, Inside the scanner with the Scanner OFF, and Scanner ON; (**B**) Time-frequency comparison of checkerboard and rest blocks over 2 s epochs across conditions; (**C**) Permutation subtraction of Checkerboard and Rest. Statistically significant regions are outlined by black lines. Natably, the frequency range around 12 and 24 Hz are statistically significant across all conditions; (**D**) Topographic plots comparing Rest and Checkerboard condition, and their difference, across three conditions.

Figure 2 shows the comparison of EEG during the checkerboard experiment across the three scan settings. Figure 2A shows the signal power over 2 s epochs during the checkerboard and rest task averaged across subjects at the Oz electrode. Comparing the checkerboard versus rest condition, the checkerboard shows peaks at 12 Hz and 24 Hz, representing the driving frequency of the checkerboard and its harmonic, respectively. When moving into the scanner environment, the power of the checkerboard is reduced. Nonetheless, peaks at 12 Hz and 24 Hz are still visible in the Scanner OFF and Scanner ON settings. The Scanner ON setting also contains a dip at 18 Hz in both rest and checkerboard conditions. This dip denotes residual artifacts that are left behind in the signal after the gradient artifact removal step. When looking at the frequency over the duration of the 2 s epoch, the difference between the rest and checkerboard condition is evident with the 12 Hz driving frequency and the 24 Hz harmonic appearing across the entire epoch (Fig. 2B). As shown in Fig. 2C,these differences are statistically significant for the three settings. While these plots focus on the Oz electrode, the signal power also extends to other electrodes in the occipital region (Fig. 2D).

**Fig. 3:**
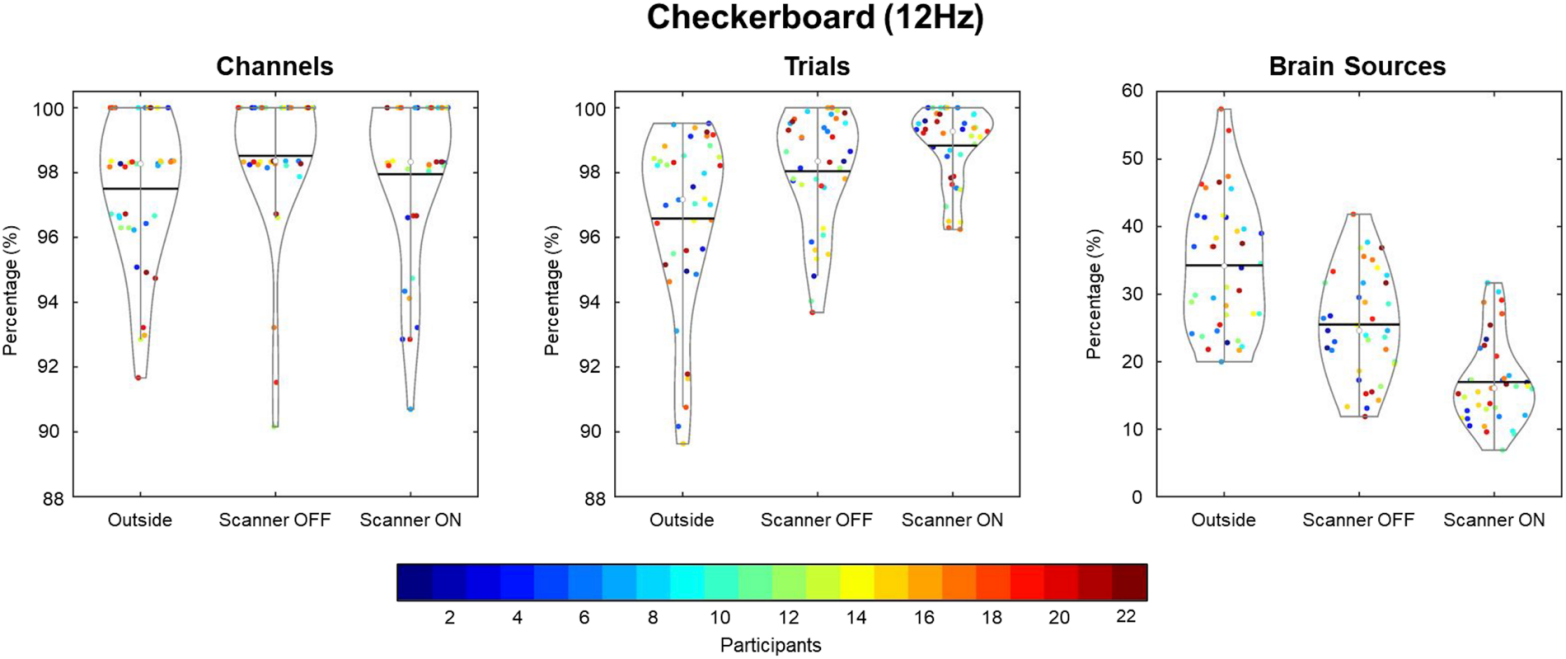
EEG data quality comparison for flickering checkerboard task across participants. Three quality metrics (percentage of good channels, percentage of good trials, and percentage of ICA brain sources) were assessed across scan conditions. For channels and trials, the data quality was high across all scan conditions, denoting high quality of EEG data.

**Fig. 4:**
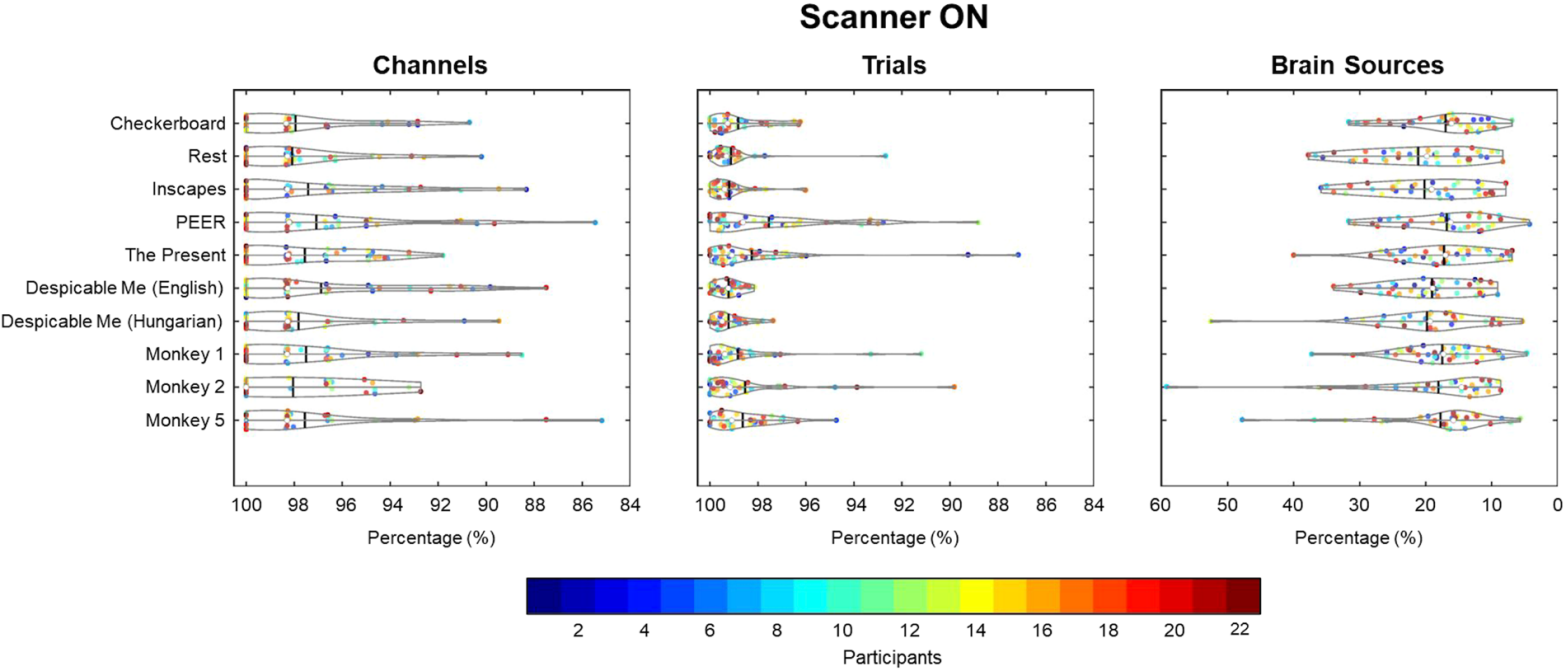
EEG data quality comparison inside scanner. Three quality metrics (percentage of good channels, percentage of good trials, and percentage of ICA brain sources) were assessed across scan conditions. For channels and trials, the data quality was high across all scan conditions, denoting the maintenance of high quality EEG data across the imaging session.

Three quality assessment metrics were computed for each raw EEG dataset: percent of “good” channels, percent of “good” trials, and the number of independent components (ICs) related to brain source activity as a percentage of the total number of ICs. As shown in Fig. 3, data quality was high across all subjects for the percentage of good channels and trials for the checkerboard task. Although the data quality was highest in the Outside setting, high percentages were found for channels and trials in the Scanner OFF and Scanner ON settings. Similarly, as seen in Fig. 4, the percentage of good channels and trials was high across tasks, denoting the stability of data quality during the scan session. The percent of putative brain sources based on ICs classification was lower for the Scanner ON setting compared to the other two settings. Due to the increase in noise sources in the Scanner OFF (e.g., pulse artifact) and Scanner ON (e.g., gradient artifact) settings, the percentage of ICs related to brain sources is expected to decrease. As shown in Fig. 4, the quality of the EEG data is stable across scan settings.

### fMRI data quality

**Fig. 5:**
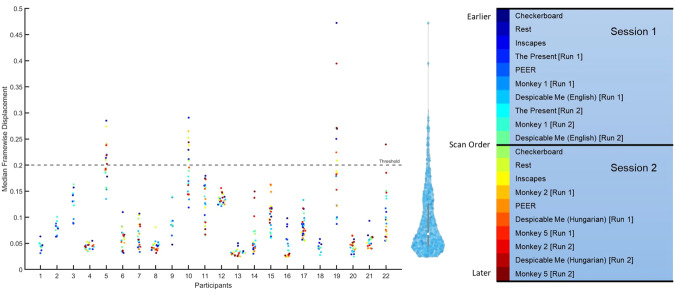
Median framewise displacement for fMRI data across the 22 participants in the study. The median framewise displacement was measured for each scan across both sessions and plotted for each participant. Scans with a value above 0.2 were considered high motion, indicated as points about the dotted threshold line. To the right of the plot, is the distribution of all scans. As seen in the distribution, participant compliance was good and most scans had low motion as measured by median FD.

To assess the quality of fMRI data, median framewise displacement (FD) was measured for all scans. As shown in Fig. 5, the median FD was for every fMRI scan; scans with a value above 0.2 were considered high motion. To determine if there was an ordering effect, scan sessions were color coded to determine if participants moved earlier or later in the scan. Most subject data were below the 0.2 threshold (93% of scans), and there was no pattern of ordering across participants.

**Fig. 6:**
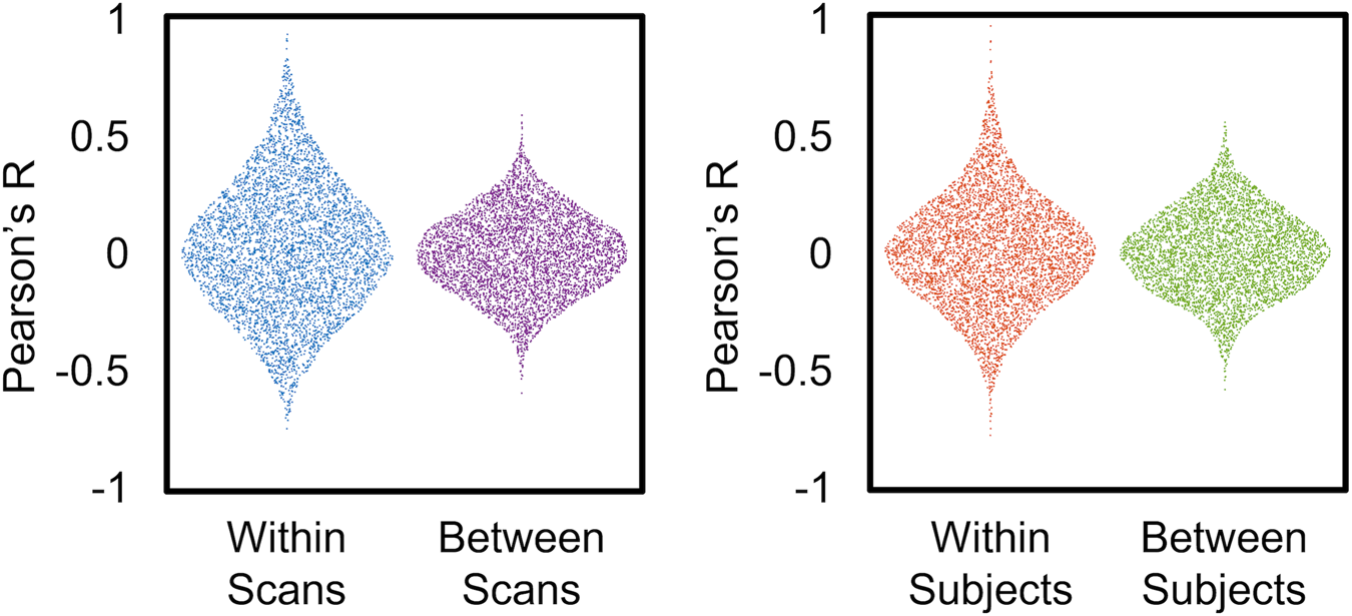
Distributions of correlation coefficients comparing within scans and between scans from the same participant, and within subject and between subject scans across all participants. When viewing a single participant, the distribution of correlation coefficients is broader (i.e., a longer tailed distribution) within scan compared to correlations between scans, reflecting stronger intrascan correlation coefficients. Similarly, the within subject correlation coefficients were stronger within participant compared to correlations between subjects.

For the checkerboard experiment, we looked at the correlation between ROIs within and between subjects and across scans (Fig. 6). The distributions for within-scan and within subjects showed a broader distribution of values, with higher correlations for within-scan and within subject distributions.

### Multimodal quality data correlations

**Fig. 7:**
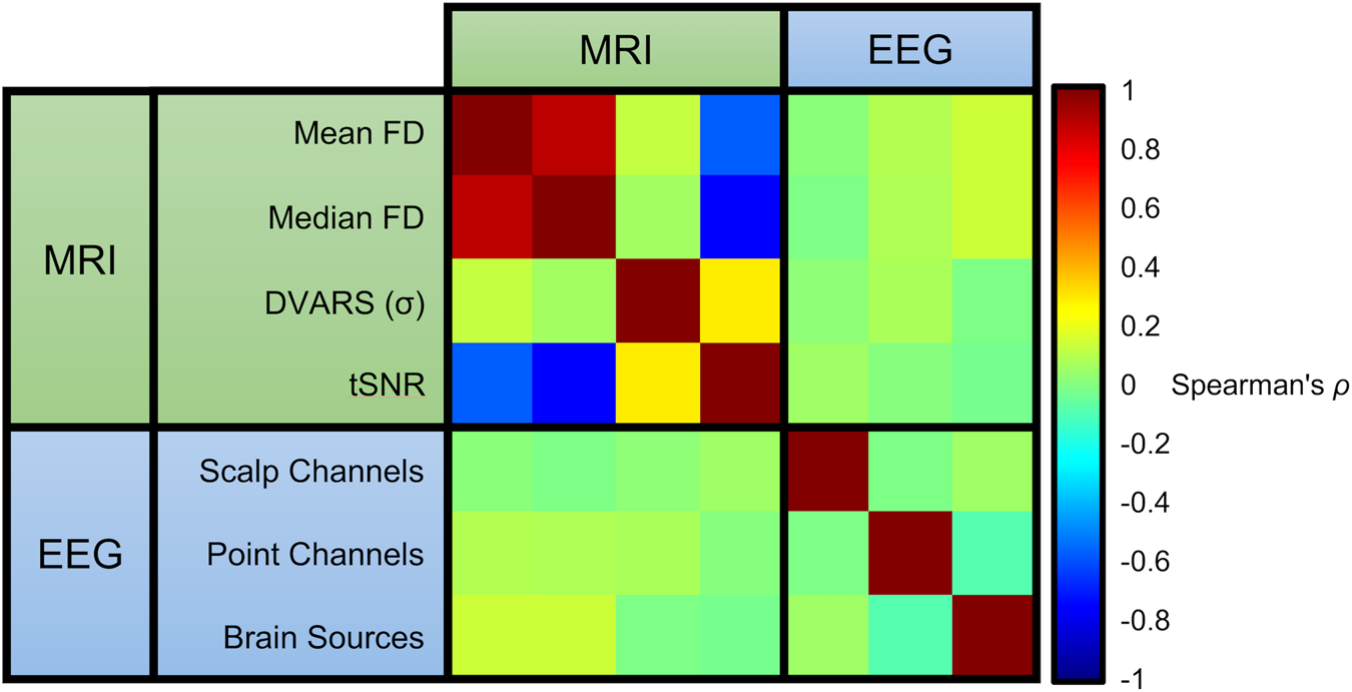
Correlation between MRI and EEG data. As expected, there is strong positive correlation between measures of mean and median FD; likewise, there is a strong negative correlation between measures of FD and tSNR. In contrast, there appears no association among EEG measures within modality and with fMRI quality measures.

Values for EEG and MRI data were compared within and between modalities across several quality metrics: mean FD (framewise displacement), median FD, DVARS (temporal derivative of time courses), and tSNR (temporal signal-to-noise ratio) for MRI; channels, trials, and brain sources for EEG (Fig. 7). Using Spearman’s *ρ* between each modality, shows a strong positive correlation between mean and median FD, and a strong negative correlation between FD measures and tSNR. A weak correlation was found between DVARS and tSNR, but no association was found with DVARS and other measures. For EEG measures, there was no correlation between the different quality measures. Moreover, there was no correlation between quality measures between imaging modalities.

### Multimodal data integration

**Fig. 8:**
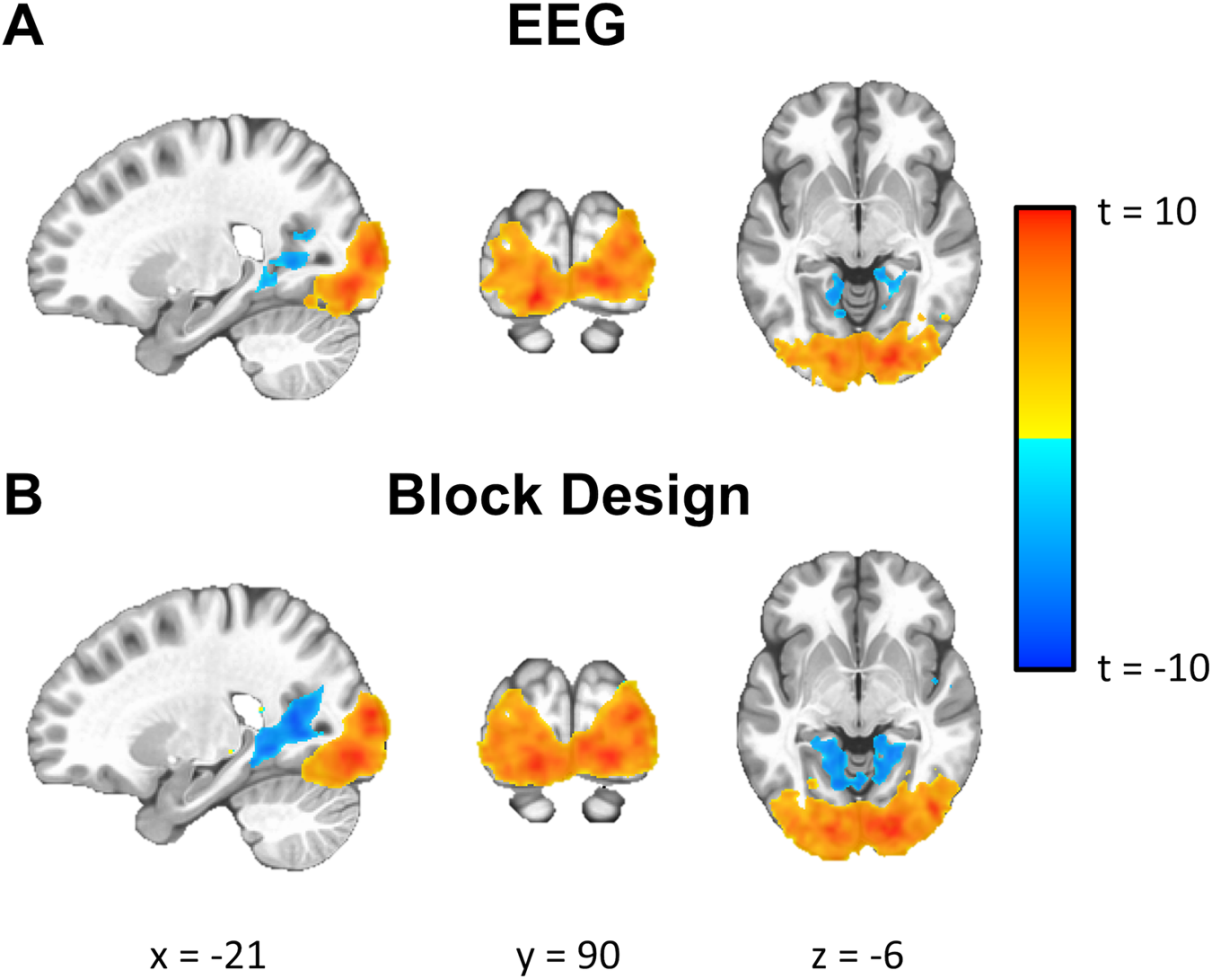
Checkerboard task group activation map either using the (**A**) group average EEG signal from Oz as a regressor or (**B**) a block design to generate the hemodynamic response function.

As a test for multimodal data integration, we evaluated whether we could use the EEG signal to predict the hemodynamic response in the fMRI data. Specifically, after preprocessing, the EEG signal from Oz signal was averaged across participants for the checkerboard experiment. This signal was then bandpass filtered (20th order IIR filter between 11 Hz and 13 Hz), modulated, and convolved with an ideal hemodynamic response function (using a gamma variate function). This signal was used as a regressor for each participant to map out the BOLD activity. A one-sample t-test was performed to calculate a group activity map (Fig. 8A). For comparison purposes, a regressor based on an ideal block design convoluted a gamma variate function was also calculated to look at the group-level activity (Fig. 8B). Activity maps shown in Fig. 8 indicate that both approaches generate a similar level of activity in the occipital lobe.

### Technical challenges

Collecting EEG and fMRI simultaneously requires several methodological considerations. While EEG and fMRI have a long-established history, collecting EEG inside the MRI scanner is challenging for several technical reasons. The main problem encountered when collecting a functional recording is the generation of artifacts from various sources. The main artifact arises from the gradient artifact generated during echo-planar imaging (EPI), which induces changes in the magnetic field^55^. Another source of noise arises from the scanner environment. While not a problem in all scanners, vibrations from the helium compressor in Siemens Trio and Verio scanners introduce artifacts into the EEG signal^56^; these vibrations induce non-stationary artifacts that contaminate the EEG signal. Yet another source of noise is caused by the pulsation of arteries in the scalp that cause movement in EEG electrodes and generation voltage. The ballistocardiogram (BCG) signal captures the ballistic forces of blood in the cardiac cycle^4,5^ and becomes more pronounced as the magnetic field strength increases^6^. In addition to a more pronounced signal, the ECG signal can impact data collection and preprocessing. In some cases, the pronounced ECG signal leads to saturation of the signal during the MRI scan sequence. Consequently, this saturation causes signal clipping that impedes QRS detection and pulse artifact removal methods during preprocessing. In this data release, there are occasions of signal clipping of the ECG channel. For participants where QRS detection of the ECG channel failed, one method used in this study was to perform QRS detection on every EEG channel and select the channel containing the mode of the detected QRS complexes. From this channel, the median template is created and applied across channels for pulse artifact removal.

To address these numerous sources of noise, there are also techniques. Gradient artifacts can be minimized by modifying the configuration or layout of EEG leads^57^ or placement of the head in the coil^58^. To remove the gradient field artifact, we use the MR clock to record the scanner trigger at every TR^59,60^. Using a template artifact subtraction method^61^, the gradient artifact is recorded at each TR onset and averaged to create a template. The template is then subtracted from the signal to produce a clean signal. In this study, we used the MRIB plug-in for EEGLAB, provided by the University of Oxford Centre for Functional MRI of the Brain (FMRIB), to regress the MRI gradient artifact^37,38^. For noise induced by the helium compressor, there are methods for recording and regressing this motion induced artifact^7,62^; however, in our experiments, the simplest method for removing this artifact was to turn off the helium compressor during simultaneous recordings. While there is a risk of helium boiling off as the temperature rises in the scanner, this can be addressed by having shorter scan sessions. In our study, the temperature of the cooling system did not fluctuate, which would impact cryogen loss. While shorter scans are ideal, we collected data for upwards of 2 hours without issue.

Another factor found to impact EEG data quality was signal clipping, which often appears in the ECG channel during simultaneous recordings. In our simultaneous EEG-fMRI recordings, cable and amplifier placement inside the scanner affected EEG data quality. For cable placement, several factors must be taken into consideration. When scanning, researchers must ensure their setup minimizes loops, cables should run along the center of the bore, and the connected amplifier should be placed at the center of the bore to ensure better data quality^55^. Excessive bends or loops in wires can induce currents in the cables, thus introducing artifacts into the EEG signal. Another way to reduce artifacts is to reduce cable length between the EEG cap and connected amplifiers. All major scanner vendors offer head coils that are designed with a channel for EEG cables that lie directly above a participant’s head^63^. In addition, cables that are bundled produce fewer artifacts than ribboned cables^64^. In our experiments, the head coil did not contain a channel for EEG cables and a ribboned cable was used to connect the cap and amplifier. To reduce artifacts, EEG cables were run through the head coil above the participant’s head and taped along the center of the bore to minimize movement and to ensure an optimal position in the scanner.

## Data Availability

Code for presenting task stimuli and naturalistic stimuli, along with code to preprocess EEG and fMRI imaging data, is available on GitHub (https://github.com/NathanKlineInstitute/NATVIEW_EEGFMRI). Additionally, the videos used for naturalistic stimuli will also be made available through the GitHub repository.
